# Quantitative proteomic analysis shows differentially expressed HSPB1 in glioblastoma as a discriminating short from long survival factor and NOVA1 as a differentiation factor between low-grade astrocytoma and oligodendroglioma

**DOI:** 10.1186/s12885-015-1473-9

**Published:** 2015-06-25

**Authors:** Marcela Gimenez, Suely Kazue Nagahashi Marie, Sueli Oba-Shinjo, Miyuki Uno, Clarice Izumi, João Bosco Oliveira, Jose Cesar Rosa

**Affiliations:** 1 Department Molecular and Cell Biology and Protein Chemistry Center, CTC-Center for Cell Therapy-CEPID-FAPESP-Hemocentro de Ribeirão Preto, Ribeirão Preto Medical School, University of São Paulo, São Paulo, Brazil; 2 Department of Neurology, São Paulo Medical School, University of Sao Paulo, Av. Bandeirantes, 3900-14049-900 Ribeirão Preto, São Paulo Brazil; 3 Instituto de Medicina Integral Prof. Fernando Figueira-IMIP, Pernambuco, Brazil; 4 Center for Studies of Cellular and Molecular Therapy (NETCEM) University of Sao Paulo, São Paulo, Brazil

**Keywords:** Glioma, Network analysis, Isobaric tag, Cancer proteomics, Biomarkers

## Abstract

**Background:**

Gliomas account for more than 60 % of all primary central nervous system neoplasms. Low-grade gliomas display a tendency to progress to more malignant phenotypes and the most frequent and malignant gliomas are glioblastomas (GBM). Another type of glioma, oligodendroglioma originates from oligodendrocytes and glial precursor cells and represents 2–5 % of gliomas. The discrimination between these two types of glioma is actually controversial, thus, a molecular distinction is necessary for better diagnosis.

**Methods:**

iTRAQ-based quantitative proteomic analysis was performed on non-neoplastic brain tissue, on astrocytoma grade II, glioblastoma with short and long survival and oligodendrogliomas.

**Results:**

We found that expression of nucleophosmin (NPM1), glucose regulated protein 78 kDa (GRP78), nucleolin (NCL) and heat shock protein 90 kDa (HSP90B1) were increased, Raf kinase inhibitor protein (RKIP/PEBP1) was decreased in glioblastoma and they were associated with a network related to tumor progression. Expression level of heat shock protein 27 (HSPB1/HSP27) discriminated glioblastoma presenting short (6 ± 4 months, *n* = 4) and long survival (43 ± 15 months, *n* = 4) (*p* = 0.00045). Expression level of RNA binding protein nova 1 (NOVA1) differentiated low-grade oligodendroglioma and astrocytoma grade II (*p* = 0.0082). Validation were done by Western blot, qRT-PCR and immunohistochemistry in a larger casuistry.

**Conclusion:**

Taken together, our quantitative proteomic analysis detected the molecular triad, NPM1, GRP78 and RKIP participating together with NCL and HSP27/HSPB1 in a network related to tumor progression. Additionally, two new important targets were uncovered: NOVA1 useful for diagnostic refinement differentiating astrocytoma from oligodendroglioma, and HSPB1/HSP27, as a predictive factor of poor prognosis for GBM.

**Electronic supplementary material:**

The online version of this article (doi:10.1186/s12885-015-1473-9) contains supplementary material, which is available to authorized users.

## Background

Gliomas are the most frequent primary tumors of the central nervous system, accounting for more than 60 % of all brain tumors, and comprise of astrocytomas, oligodendrogliomas, oligoastrocytomas, and ependinomas [1]. Among them, glioblastoma (GBM-grade IV astrocytoma) is the most malignant glioma and despite continuous efforts, the median survival still remains around 15 months after the establishment of diagnosis and the standard care with radiation therapy and chemotherapy with temozolamide [2]. The main study design concerning GBM has aimed to uncover specific drugable targets in signaling pathways with impact in the tumorigenic process and in the extension of overall survival time [3]. In this context, we have recently described two proteins, nucleophosmin (NPM1) and RKIP, involved in RAS/RAF/MAPK and PI3K/AKT/mTOR pathways [4]. We have also shown that NPM1 knockdown sensitized GBM cell lines to cell death after treatment with temozolamide [5]. Moreover, when NPM1 expression was silenced, expression of GRP78, a member of the heat shock protein 70 involved in protein unfold response, was concomitantly decreased. GRP78 expression was high in GBM, and correlated to cell migration [6]. In the present study we have compared the protein expression profiles of GBM cases presenting short and long survival time, and astrocytoma and oligodendroglioma of different grades of malignancy to further understand the mechanisms of tumor aggressiveness.

Another strategy to understand the rules governing the aggressive behavior of gliomas is to compare astrocytoma to oligodendroglioma, where the latter type of glioma presents a less aggressive clinical evolution. Five and 10 years survival rates for oligodendroglioma are 78 and 51 %, respectively, whereas among astrocytoma they are 65 and 31 %, respectively [7, 8]. This survival rate difference is due partially to a better response of oligodendroglioma to chemotherapy, including temozolomide or PCV- procarbazin, 1-(2-cloroethyl)-3-cyclohexil-L-nitrosurea and vincristin [9–14] and to radiation therapy [15, 16]. Therefore, further analysis of differential protein profiles of these glioma types may help to: 1) refine the histopathologic diagnosis, currently based mainly in morphologic characteristics, with large interobserver variability [17, 18], and 2) detect molecular targets that may explain the difference of clinical outcome between low grade astrocytoma and oligodendroglioma.

In this study, we took advantage of isobaric tags for relative and absolute quantification (iTRAQ-8plex) to investigate the proteome related to tumor progression and aggressiveness comparing a set of astrocytoma grade II to oligodendroglioma grade II, and a set of GBM cases presenting short survival (6 ± 4 months, *n* = 4) to GBM cases with long survival (43 ± 15 months, *n* = 4). We have succeeded in uncovering differential protein profiles between these compared sets, highlighting two targets, HSPB1/HSP27 and NOVA1, related to tumor progression and differentiation. Both selected targets were further validated at mRNA expression levels by quantitative PCR, and protein expression and intracellular localization by immunohistochemistry in an independent casuistry of human glioma samples.

## Methods

### Tissue processing

Tissue samples from tumors were collected during surgery and stored at −80 °C. Tissue samples were micro-dissected in order to remove areas of necrosis, cellular debris and any non-neoplastic tissue prior to protein, DNA and RNA extraction. The tumor area of interest was concomitantly collected for pathological diagnosis and grade stratification according to the latest WHO classification of CNS tumors by two independent pathologists. The tumors were graded as AST II astrocytoma grade II (AST II), glioblastomas (GBM) and oligodendrogliomas grade II (OLI II) and oligodendrogliomas grade III (OLI III). GBMs were divided in two subgroups based on patients’ overall survival time after diagnosis as GBM of short survival (GBM-SS, 6 ± 4 months, *n* = 4) and long survival (GBM-LS, 43 ± 15 months, *n* = 4). Non-neoplastic brain tissues (NN, mean age at surgery, 29 ± 7 years, *n* = 4) were obtained from individuals submitted to temporal lobe resection for epilepsy surgery and examined by a pathologist who confirmed the abundance of astrocytic cells in the resected tissue. Four samples for each group were pooled and analysed by the proteomic approach (ASTII mean age at diagnosis, 33 ± 7 years; GBM-SS 48 ± 23 years; GBM-LS 48 ± 18 years; OLI II 42 ± 16 years and OLI III 48 ± 15 years). An independent casuistry comprised of 22 (NN), 23 (AST I), 26 (AST II), 18 (AST III), 83 (AST IV or GBM), 25 (OLI II), and 26 (OLI III) was analyzed at the validation step by qRT-PCR for the selected targets. All samples were collected during surgical procedures by the Neurosurgery Group of the Department of Neurology at the Hospital das Clinicas of School of Medicine of São Paulo, University of Sao Paulo, Brazil from 2000 to 2008 and the follow-up of cases are being carried out to date. This study was approved by the Brazilian National Bioethics Commission (CONEP), and by the Ethics Committee of the Medical School of Ribeirao Preto and School of Medicine of São Paulo of the University of Sao Paulo. Written consent was obtained from each patient authorizing the use of their tissues in the present investigation.

### Tumor protein extraction

Tissue samples were mechanically homogenized in lysis buffer containing 30 mM Tris-HCl pH 7.5, 150 mM NaCl, 1 % Triton X-100, 10 % glycerol and a protease inhibitor cocktail. The cell lysates were centrifuged at 20,000 g for 30 min, the supernatants were precipitated with 20 % trichloroacetic acid and washed three times with cold acetone. Electrophoresis buffer (200 µL) containing 10 mM Tris base, pH 9.0, 7 M urea, 2 M thiourea, 65 mM DTT and 4 % CHAPS was added to each pellet. Proteins pellets were then submitted to three cycles of 5 min each in an ultrasound bath (UltraSonic Clear 750, UNIQUE) centrifuged and supernatant were kept for protein concentration determination.

### Sample preparation and iTRAQ labeling

Each protein extract of tumor and non-neoplastic tissue were quantified by the method of Bradford [19]. Twenty five μg of each patient sample was pooled to normalize 100 μg total protein for each category. Additional file 1: Figure S1 describes a schematic experimental approach. Pooled samples were mixed with 6× volume of cold acetone (−20 °C) and incubated for 60 min at −20 °C. The proteins pellets were reconstituted according to manufacturer’s protocol (Applied Biosystems, Framingham, MA, USA). Briefly, proteins pellets were re-suspended into 20 μL of dissolution buffer (0.5 M triethylammonium bicarbonate), 1 μL denaturant (2 % SDS), and 2 μL reducing reagent (50 mM tris-(2-carboxyethyl) phosphine). Free cysteine was blocked by adding 1 μL of 200 mM methyl methanethiosulfonate in isopropropanol. Sequencing grade modified trypsin was from Promega (Madison, WI) and was reconstituted with deionized water at 1 μg/μL concentration. In each vial 10 μL of trypsin solution was added and incubated overnight (18 h) at 37 °C. Reagents of 8plex iTRAQ were allowed to reach room temperature and then reconstituted with 50 μL of isopropanol. Each label reagent was mixed with the corresponding protein digest and incubated at room temperature for 2 h. Samples were pooled into a new vial and dried in SpeedVac (Savant Inc, New York, NY). After reconstituted with 0.1 % formic acid (FA), the digest was desalted on a Waters Oasis HLB column and eluted with 60 % acetonitrile (ACN)/ 0.1 % FA. Eluted peptide mixture was dried.

### Strong cation exchange fractionation (SCX)

The sample was reconstituted with 100 μL SCX buffer A (10 mM KH_2_PO_4_, 20 % ACN, pH2.7) and separated on a PolyLC Poly-sulfoethyl-A column (200x2.1 mm, 5 μm, 200 Å) with a linear 200 μL/min gradient of 0-70 % buffer B (10 mM KH_2_PO_4_, 20 % ACN, 500 mM KCl, pH2.7) in 45 min on an Agilent 1200 LC device with Chemstation B.02.01 control software. Fractions were collected each minute and eventually pooled into 20 fractions. The fractions were desalted, eluted, and dried as described above using Waters Oasis HLB column.

### *Mass spectrometry*

The samples were reconstituted with 0.1 % formic acid. Liquid chromatography was performed on an Eksigent nanoLC-Ultra 1D plus system (Dublin, CA). Peptide digest was first loaded on a Zorbax 300SB-C18 trap (Agilent, Palo Alto, CA) at 6 μL/min for 5 min, then separated on a PicoFrit analytical column (100 mm long, ID 75 μm, tip ID 10 μm, packed with BetaBasic 5 μm 300 Å particles, New Objective, Woburn, MA) using a 40-min linear gradient of 5-35 % ACN in 0.1 % FA at a flow rate of 250 nL/min. Mass analysis was carried out on an LTQ Orbitrap Velos (Thermo Fisher Scientific, San Jose, CA) with data-dependent analysis mode, where MS1 scanned full MS mass range from m/z 300 to 2000 at 30,000 mass resolution and six HCD MS2 scans were sequentially carried out at resolution of 7500 with 45 % collision energy, both in the Orbitrap.

### Database search and quantitative data analysis

MS/MS spectra from 20 fractions were searched against the Swiss Prot (Swiss Institute of Bioinformatics) database, taxonomy Homo sapiens (human) using Mascot software (Matrix Science, London, UK; version 2.3), with precursor mass tolerance at 20 ppm, fragment ion mass tolerance at 0.05 Da, trypsin enzyme with 2 miscleavages, methyl methanethiosulfonate of cysteine and iTRAQ 8plex of lysine and the n-terminus as fixed modifications, and deamidation of asparagine and glutamine, oxidation of methionine and iTRAQ 8plex of tyrosine as variable modifications. The resulting data file was loaded into Scaffold Q+ (version Scaffold 4.3.0, Proteome Software Inc., Portland, OR) to filter and quantitate peptides and proteins. Peptide identifications were accepted at 80.0 % or higher probability as specified by the Peptide Prophet algorithm [20] and a false discovery rate (FDR) of less than 1 %. Protein identifications were accepted at 95.0 % or higher probability and contained at least 2 identified peptides with FDR less than 1 %. Protein probabilities were assigned by the Protein Prophet algorithm [21]. Proteins that contained similar peptides and could not be differentiated based on MS/MS analysis alone were grouped to satisfy the principles of parsimony. Peptides were quantified as the centroid reporter ion peak intensity, with minimum of 5 % of the highest peak in the spectrum. Intra-sample channels were normalized based on the median ratio for each channel across all proteins. Isobaric tag sample was normalized by comparing the median protein ratios for the reference channel. Quantitative protein values were derived from only uniquely assigned peptides. Protein quantitative ratios were calculated as the median of all peptide ratios. Standard deviations were calculated as the interquartile range around the median. Quantitative ratios were log_2_ normalized for final quantitative testing.

### *Western blot*

The samples were diluted in NuPAGE SDS Sample buffer (Invitrogen NP0007) and the SDS-PAGE was performed using NuPAGE Novex Bis-Tris Mini Gels 4–12 %. SDS-PAGE gels were electrobloted in iBlot Device and the membranes were incubated with primary antibodies HSPB1/HSP27 and HSP90B1(GRP94) from Cell Signaling Technology; NPM and RKIP from Zymed-Invitrogen; NCL and β-actin from Santa Cruz Biotechnology; NOVA-1 from Sigma-Aldrich. The same source of antibodies HSPB1 and NOVA1 were used for immunohistochemistry.

### RNA extraction and cDNA synthesis

Total RNA was extracted from each tissue using the RNeasy Mini Kit (Qiagen, Hilden, Germany). RNA quantification and purification was determined by measuring absorbance at 260 and 280 nm. A260/A280 ratios in the 1.8–2.0 range were considered to indicate a satisfactory level of purity. Denaturing agarose gel electrophoresis was used to assess the quality of the samples. cDNA synthesis was performed by reverse transcription of 1 μg total RNA previously treated with one unit of DNase I (FPLC-pure, GE Healthcare, Piscataway, NJ,) using random and oligo(dT) primers, RNase inhibitor, and SuperScript III (Life Technologies) according to the manufacturer’s recommendations.

### Quantitative real-time PCR (qRT-PCR)

For qRT-PCR, quantitative data were normalized relative to the internal housekeeping control genes hypoxanthine phosphoribosyltransferase 1 (*HPRT*), beta-glucuronidase (*GUSB*), and TATA-box binding protein (*TBP*) [22]. The geometric mean of the housekeeping genes was used for the analysis of relative expression of tissue samples. Primer sequences were as follows (5′– 3′): *HSPB1* F: GGACGAGCTGACGGTCAAGA, *HSPB1* R: CGGGAGATGTAGCCATGCT, *NOVA1* F: GGAGCCACCATCAAGCTGTCTA, *NOVA1* R: TCAGTGCTTCAACCGTTCCCT, *HPRT* F: TGAGGATTTGGAAAGGGTGT, *HPRT* R: GAGCACACAGAGGGCTACAA, *GUSB* F: AAAATACGTGGTTGGAGAGCTCATT, *GUSB* R: CCGAGTGAAGATCCCCTTTTTA, *TBP* F: AGGATAAGAGAGCCACGAACCA, and *TBP* R: CTTGCTGCCAGTCTGGACTGT synthesized by IDT. Sybr Green I amplification mixtures (12 μL) contained 3 μL cDNA, 6 μL 2 × Power Sybr Green I Master Mix (Applied Biosystems, Foster City, CA), and forward and reverse primers at final concentrations of 200–400 nM. Reactions were run on an ABI 7500 Real-Time PCR System (Applied Biosystems). The cycling conditions were: incubation at 50 °C for 2 min to activate UNG, initial denaturation at 95 °C for 10 min, and 40 cycles of 15 s each at 95 °C and at 60 °C for 1 min. DNA melting curve analysis showed a single peak for all genes. The 2^−ΔΔCT^ equation was applied to calculate the relative expression [23]. For the relative expression analysis of GBM cases, the mean of control non-neoplastic brain samples was used as calibrator.

### *Immunohistochemistry*

For immunohistochemical detection of HSPB1 and NOVA1, tissue sections were routinely processed and subjected to antigen retrieval. Briefly, slides were immersed in 10 mM citrate buffer, pH 6.0 and incubated at 122 °C for 3 min using an electric pressure cooker (BioCare Medical Walnut Creek, CA). Specimens were then blocked and further incubated with a mouse monoclonal antibody raised against human HSPB1 and NOVA1 at a final dilution of 1:100 at 16-20 °C for 16 h. The reaction was developed using a Novolink commercial kit (Novocastra, New Castle, UK) at room temperature using diaminobenzidine, and Harris hematoxylin for nuclear staining. All prepared slides were independently analyzed by two observers, and the positive reaction was quantitated for HSPB1 and NOVA1 as the percentage of positive cytoplasm/nuclei cells: zero (0), when no positivity was detected; 1, when up to 25 % of positive cells were present; 2, for 26-50 % of positive cells; 3, for 51-75 % of positive cells, and 4, for over 76 % of positive cells.

### *Statistical analysis*

The statistical analysis of HSBP1 and NOVA1 expression by qRT-PCR in astrocytomas, oligodendrogliomas and non-neoplastic tissues was performed by Kruskal-Wallis and Mann-Whitney tests as well as the proteomic profiling through statistical package included in Scaffold v.4.3.0 software, blocked *t*-test and ANOVA for categories (p-value, ASTRO, OLI or ASTRO/OLI) (Proteome Software, Inc, Portland, Oregon). Discrimination of variables was calculated by the receiver operator characteristic (ROC) curve utilizing area under curve and asymptotic significance. The continuous variables were categorized through a curve using ROC the value with the best sensitivity and specificity. Differences in gene and protein expressions were considered to be statistically significant at *p* < 0.05.

## Results

### Identification of proteins differentially expressed in gliomas using isobaric tags for relative and absolute quantification (iTRAQ)

**Fig. 1:**
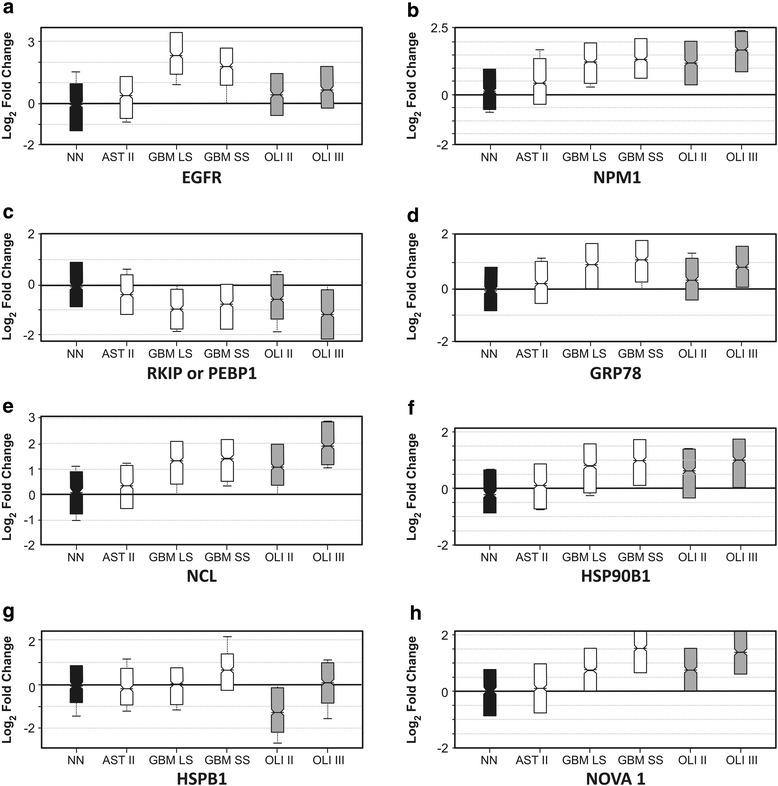
Proteins differentially expressed in astrocytomas and oligodendrogliomas. Panels A to H in the figure represent the differentially expressed proteins by log2 fold change for each of the selected proteins which are calculated dividing all the peaks by the average of the isobaric tag peak intensities appearing in the spectra included in NN category and that the spread shown for the log2 fold change of NN illustrates the variation of the isobaric tag peak intensities within the reference label in respect to their average

Proteomic analysis using iTRAQ isobaric tags was performed using pool of samples from astrocytoma grade II (AST II), glioblastoma (GBM) sub-grouped into cases presenting short and long survival after diagnosis (GBM-SS, 6 ± 4 months, *n* = 4 and GBM-LS43 ± 15 months, *n* = 4, respectively), oligodendroglioma grade II (OLI II) and oligodendroglioma grade III (OLI III). The proteins were selected and quantified in Scaffold software v.4.3.0 (Fig. 1 and Table 1). Proteins were differentially expressed when compared to non-neoplastic tissue (NN) as the ratio was above or below Log2 Fold Change (0.6 = 1.5-fold) and statistically significant between categories. The results of the following sets were compared: 1) AST II vs. GBMs, and OLI II vs. OLI III to address protein involved in tumor malignant progression; 2) GBM-SS vs. GBM-LS to address proteins involved in prognosis; 3) AST II vs. OLI II to address proteins involved in the differentiation between these two low grade gliomas with impact in tumor aggressiveness. We were able to identify 1095 proteins labeled with iTRAQ and using minimum of 2 peptides per protein (Additional file 3: Table S1 - Protein report and Additional file 4: Table S2 Peptide report), which 268 presented difference of expression in at least one group (Additional file 5: Table S3 Protein ratio). The gene ontology analysis revealed that proteins differentially expressed were mainly involved with metabolic processes, biological processes regulation and binding to proteins, RNA and nucleotides.

**Table 1:** Selected proteins from quantitative proteomic analysis of astrocytomas and oligodendriomas tumor samples (*n* = 4). Proteins are expressed as log2 fold change in relation to non-neoplastic brain tissue (NN)

Id Protein	Acc	NN	AST II	GBM SS	GBM LS	OLI II	OLI III	Spectra Count	% Seq Cov	blocked *t*-test (p-value ASTRO)^a^	blocked *t*-test (p-value OLI)^b^	blocked ANOVA test (p-value ASTRO/OLI)^c^
Epidermal growth factor receptor	EGFR	Ref	0.2	1.5	2.2	0.3	0.6	20	13.2	0.0023	0.93	<0.0001
Nucleophosmin	NPM1	Ref	0.2	1.1	1.1	0.8	1.3	14	32.3	0.0049	<0.0001	<0.0001
78 kDa glucose-regulated protein	GRP78 (HSPA5)	Ref	0.3	1.1	0.9	0.2	0.6	44	43.0	<0.0001	0.0008	0.037
Phosphatidylethanolamine-binding protein 1	PEBP1 (RKIP)	Ref	-0.4	-1.0	-1.1	-0.6	-1.4	36	79.1	<0.0001	<0.0001	<0.0001
Heat shock protein beta-1	HSPB1	Ref	0.1	1.6	0.8	0.3	0.5	9	49.8	0.00045	0.89	0.28
RNA-binding protein Nova-1	NOVA1	Ref	0.1	0.9	2.2	0.8	1.3	3	7.8	0.120	0.011	0.0082
Endoplasmin	(ENPL) HSP90B1	Ref	0.2	1.0	0.9	0.6	1.0	44	25.8	<0.0001	<0.0001	<0.0001
Nucleolin	NCL	Ref	0.2	1.3	1.1	1.0	1.9	27	25.1	<0.0001	<0.0001	<0.0001

Statistical test for ratio-based normalization of isobaric tags (Scaffold v.4.3.0) - The samples were grouped as follows:

^a^astrocytomas = NN, AST II, GBM-SS and GBM-LS

^b^oligodendroglioma = NN, OLI II and OLI III

^c^strocytoma and oligodendroglioma = NN, AST II and OLI II

### Selection and validation of proteins involved in tumor malignant progression

**Fig. 2:**
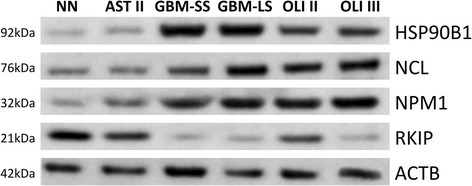
Western blot validation of differentially expressed proteins in patient pools of astrocytomas and oligodendrogliomas. The same pool of patient sample used for quantitative proteomics was used for validation by immunodetection. The assay was normalized to actin

Proteins selected as having alteration of expression and known to participate in the process of tumor progression are shown in Table 1 and Fig. 1. NPM1, RKIP/PEBP1 and GRP78 expressions were significantly distinct in GBMs and oligodendrogliomas compared to AST II and NN (*p* < 0.0001, blocked *t* test), corroborating previous data of our group [4–6]. Particularly, NPM1 expression presented correlation with tumor malignant progression as lower expressions were observed in non-neoplastic tissue, AST II and OLI II compared to the expressions of GBM and OLI III. Interestingly, phosphatidylethanolamine-binding protein 1 (PEBP1), also known as raf kinase protein inhibitor (RKIP), was decreased in high grade gliomas in relation to the non-neoplastic tissue and lower grade gliomas, as previously demonstrated by our group [4]. These reproduced data related to NMP1, GRP78 and RKIP demonstrated the consistency of the proteomics results herein presented by iTRAQ methodology. Also, EGFR was highly expressed in GBM compared to AST II, as expected, however, similar results were not observed for OLI II and III (*p* = 0.930) (Fig. 1a). Selected proteins as NPM1, RKIP, HSP90B1 and NCL were also differentially expressed among the analyzed subgroups and these levels of expression were validated by western blotting of pooled samples (Fig. 2). GRP78 was previously validated elsewhere [5, 6].

### HSPB1 (HSP27) as a predictive factor between GBM cases with short and long overall survival time

**Fig. 3:**
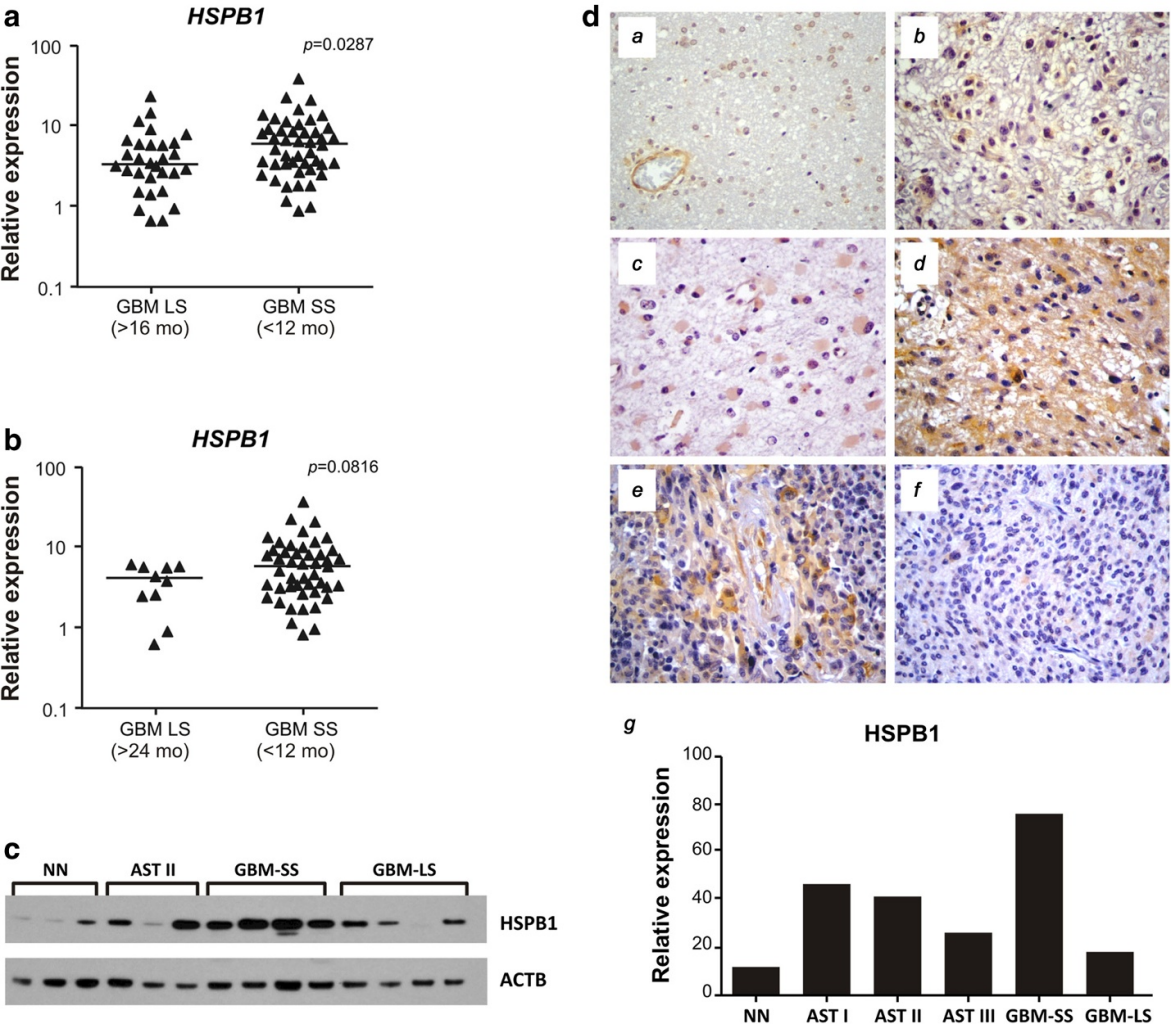
Validation of proteomic data of HSPB1 in glioma patient samples. **a** qRT-PCR of *HSPB1* gene in GBM patient samples with short survival time (<12 months) and long survival (>16 months). **b** Re-analysis of the qRT-PCR of *HSPB1* gene in samples for a longer interval between short (<12 months) and long survival patients (>24 months). Data analyses were normalized by the same set of housekeeping genes: *HPRT, GUSB, TBP* and Mann Whitney statistical test. **c** Western blot of HSPB1 in individual astrocytoma patient samples (*n* = 3 and 4); (NN = non-neoplastic tissue; AST II = astrocytoma grade II, GBM-SS = glioblastoma short survival and GBM-LS = glioblastoma long survival. **d** Immunohistochemistry (IHC) of HSPB1 in low-grade to high-grade gliomas. *a* Non neoplastic (NN); *b* Pilocytic astrocytoma (ASTI); *c* Diffuse astrocytoma (ASTII); *d* Anaplastic astrocytoma (ASTIII), and *e* Glioblastoma (GBM) short survival (5 months), and *f* GBM long survival (27 months). *g* graphical-plot of IHC relative expression of HSPB1

**Fig. 4:**
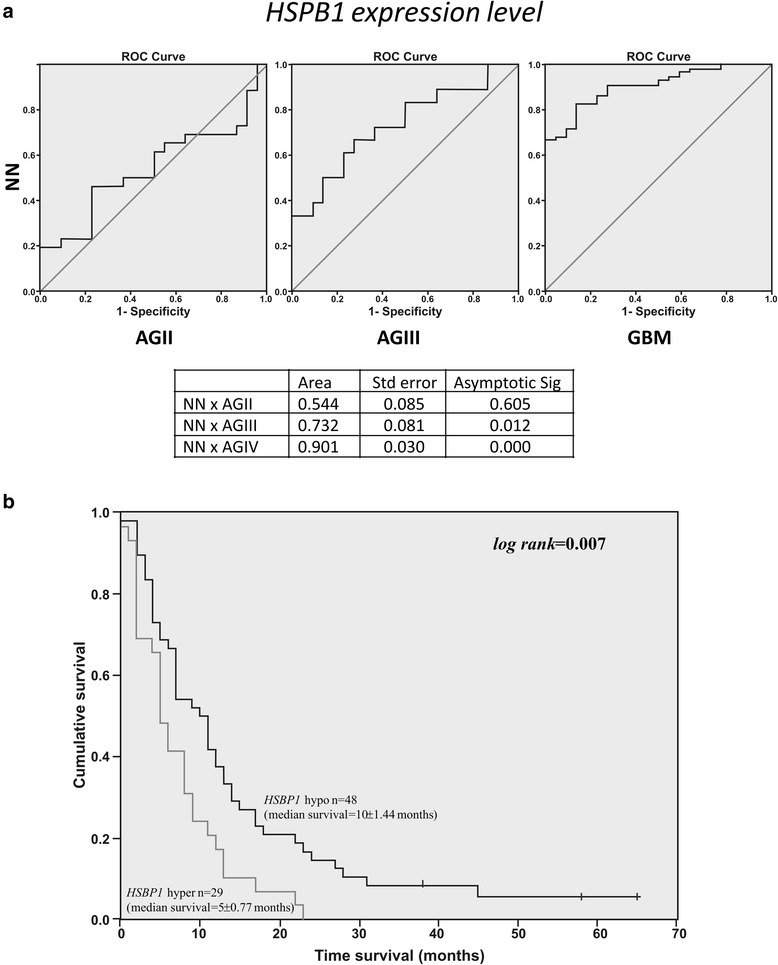
**a** ROC curves comparing *HSPB1* expression levels of NN group compared to each grade of malignant astrocytomas, grade II to IV. The values of area, standard error under the nonparametric assumption, and asymptotic significance considering null hypothesis of true area - 0.5; **b** Kaplan-Meier curves. Overall survival time of GBM cases presenting *HSPB1* expression level > 23.28 (3 fold of cut off value determined by ROC curve) (*n* = 29) compared to GBM cases presenting *HSPB1* expression level < 23.28 (*n* = 48). Log rank = 0.007

The most interesting differentially expressed protein was heat shock protein beta-1 (HSPB1) (Fig. 1g, Table 1) that was highly expressed in GBM-short survival (GBM-SS), especially when compared to GBM-long survival (GBM-LS) (*p* = 0.00045). This finding was also observed at the *HSPB1* mRNA expression level, where its expression was significantly different among GBM patients who presented less than 12 months of survival time compared to those presenting more than 16 months survival (*p* = 0.0287, Mann Whitney test). *HSPB1* expressions were still distinct when GBMs cases with less than 12 months and more than 24 months survivals were compared (*p* = 0.0816, Mann Whitney test) (Fig. 3a and b). Statistical significance would be reached increasing the number of observations of GBM cases presenting overall survival time longer than 24 months, a very rare condition. Additionally, a stepwise increase of *HSPB1* mRNA expression was observed in parallel to the increase in malignancy mainly in diffusely infiltrative astrocytomas from grade II to IV (*p* <0.05 to *p* <0.0001, Kruskal Wallis and Dunn's tests), and when these results were plotted on ROC curves, an increasing value of the area in parallel to the increment of the malignancy was observed (Fig. 4a), strongly suggesting that *HSPB1* expression level as an indicator of tumor progression. Moreover, when the overall survival times of GBM cases presenting ±3 fold cut off value of *HSPB1* expression level calculated based on the ROC curve (3×7.76 = 23.28) were compared to those presenting *HSPB1* expression level < 23.28, it resulted in a Kaplan-Meier curve with log rank of 0.007 (Fig. 4b). This finding was independent to the *IDH1* mutation status [24], according to multivariate proportional hazards analysis (Cox model), where *HSPB1* expression status (hyper and hypo expression) presented hazard ratio (HR) of 1.86 with 95 % confidence interval (CI) ranging from 1.14 ± 3.03, and *p* value of 0.012. On the other hand, *IDH1* mutation status (mutated *IDH1* compared to wild type) presented HR = 1.35, 95 % CI = 0.64 ± 2.84, *p* = 0.43. Similar analysis was not feasible to *MGMT* methylation status, as such results were available for only 51 out of 83 GBM cases due to limitation of biological sample. Nevertheless, we have previously reported no impact of *MGMT* methylation status on the overall survival time among GBM cases of this series (log rank, Mantel-Cox = 0.204) [25]. HSPB1 protein expression levels of GBM cases with short and long survival were validated individually by western blot analysis, which showed more intense immunostaining of protein in GBM cases with short survival, confirming the possible usefulness of HSPB1 as predictive factor of worse prognosis (Fig. 3c). HSPB1 protein expression was further confirmed by immunohistochemistry in astrocytoma samples, comprising pilocytic astrocytoma (grade I), low grade astrocytoma (grade II), anaplastic astrocytoma (grade III) and GBM (grade IV) with short (5 months) and long survival (27 months), and in non-neoplastic brain tissue (Fig. 3d). High abundance of HSPB1 was detected in grade IV astrocytoma, particularly in GBM-SS (5 months, Fig. 3d(*e*)) with unequivocal contrast to the weak labeling of a GBM-LS case (27 months, Fig. 3d(*f*)). Graph of immunochemistry of HSPB1 was demonstrated in Fig. 3d(*g*), showing that GBM-SS is highly positive in contrast to GBM-LS. These results of proteomics, gene and protein expressions allow to elect HSPB1 as a predictive factor of tumor aggressiveness in a restricted set of GBM cases, and it may worth further exploration as a potential therapeutic target for these specific cases.

### NOVA1 as a differentiation factor between Low grade astrocytoma and oligodendroglioma

**Fig. 5:**
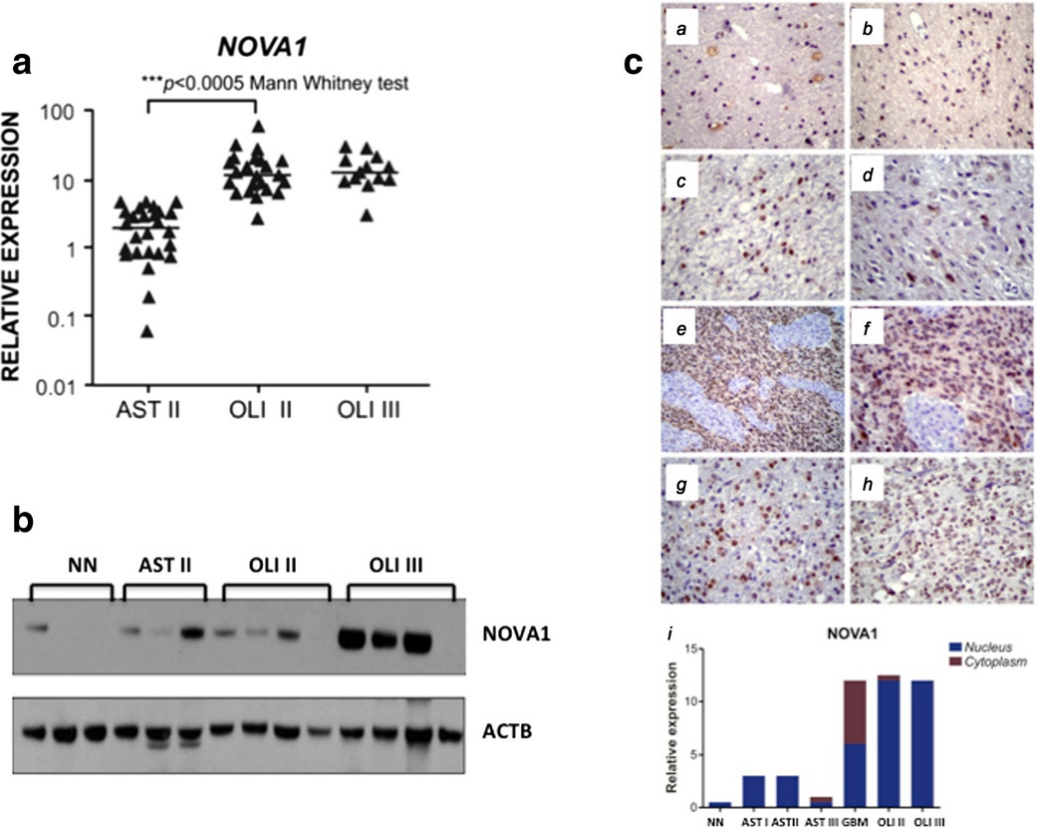
Validation of proteomic data of NOVA1 in low-grade astrocytoma and oligodendroglioma patient samples. **a** qRT-PCR of *NOVA1* gene in astrocytoma grade II (AST II) and oligodendroglioma (OLI II and OLI III). Data analyses were normalized by same set of housekeeping genes: *HPRT*, *GUSB*, *TBP* and Mann Whitney statistical test. **b** Western blot of NOVA1 in individual astrocytoma and oligodendroglioma patient samples (*n* = 3 and 4); **c** Immunohistochemistry (IHC) of NOVA1 in astrocytomas and oligodendrogliomas. *a* Non neoplastic (NN); *b* Pilocytic astrocytomas (AST I); *c* Diffuse astrocytoma (AST II); *d* Anaplastic astrocytomas (AST III), and *e* Glioblastoma (GBM, 200×) *f* Glioblastoma (GBM, 400×); *g* Oligodendroglioma II and *h* Oligodendroglioma III. *i* Graphical plot of IHC relative expression of NOVA1 distribution between nucleus and cytoplasm

RNA binding protein nova 1 (NOVA1) presented an interesting expression profile when low grade astrocytomas and oligodendrogliomas grade II were compared (Table 1, *p* = 0.0082). qRT-PCR for *NOVA1* showed a significant difference between OLI II and AST II (p < 0.0005, Mann Whitney test) (Fig. 5a). NOVA1 was validated by western blot through the analysis of patients of grade II astrocytoma and oligodendroglioma II and III individually. The results showed heterogeneity in the immunodetection of NOVA1 and at least one case, the protein was not detected, from a total of 4 samples in OLI II (Fig. 5b). However, NOVA1 immunohistochemistry was highly concordant with *NOVA1* at mRNA level and proteomic profiling, showing a major concentration of this protein in nuclei compartment (Fig. 5c). NOVA1 can be used as a molecular marker to differentiate low-grade astrocytoma from low-grade oligodendroglioma, and therefore, may be helpful for the refinement of the diagnosis currently based mainly on histopathological characteristics.

### Network analysis of molecular triad NPM1, RKIP and GRP78 using metacore

**Fig. 6:**
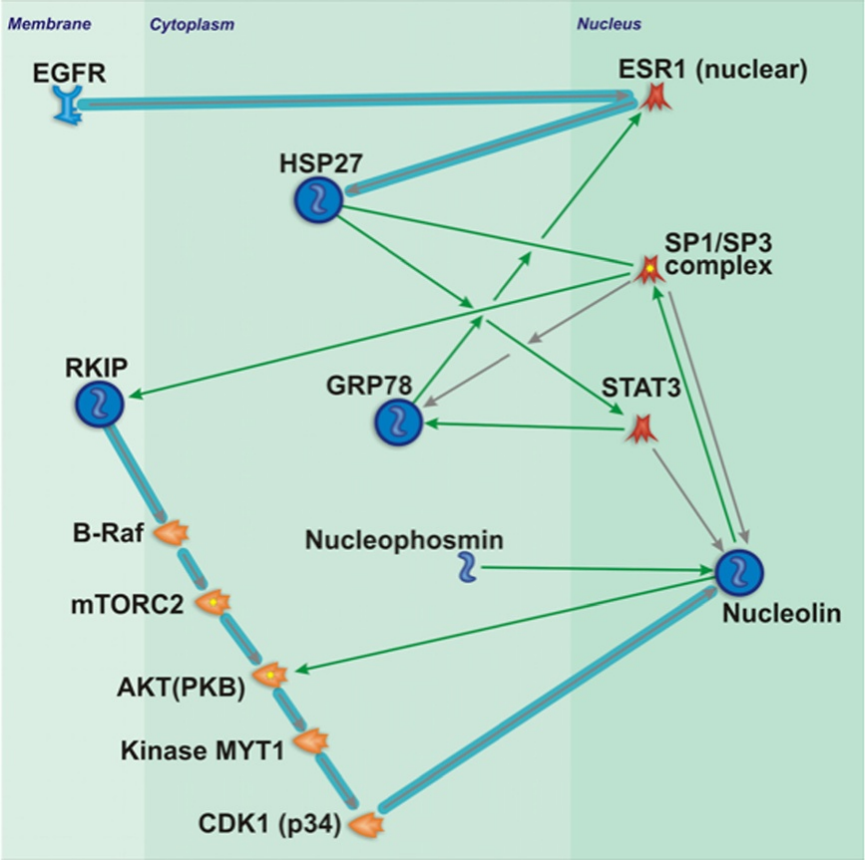
MetaCore analysis of differential modulated proteins in glioma tumors. The NPM1, RKIP, GRP78, HSP27 (HSPB1) and NCL proteins were subjected to network interactions using the MetaCore software and placed in a context of systems biology implying important findings to contribute to the progress of studies in glioma tumorigenesis and their participation in canonical pathways. See MetaCore symbol legend in Additional file 2: Figure S2

The network analysis of three molecules NPM1, RKIP and GRP78 by MetaCore program allowed the addition of two proteins in the context of systems biology, HSPB1 (HSP27) and nucleolin (NCL), interconnected to at least three transcription factors, ESR1, STAT3 and SP1, and downstream of EGFR receptor pathway, classically known as modified in GBM (Fig. 6). The results of this network analysis highlight the importance of several proteins found in this work to be altered in the tumor samples and will be discussed later in the next section.

## Discussion

One of the most productive and direct way of obtaining information about the development of diseases such as cancer, especially brain cancer, is the combination of genomic and proteomic strategies of tumor specimens taken from patients. Each tumor sample provides new markers of the disease and improves the knowledge about the biology of tumors. One of most important findings of our report is the detection of NOVA1 as differentiation factor between low-grade astrocytoma and oligodendroglioma. The clinical impact of such a diagnostic refinement based on molecular marker is relevant as astrocytoma presents more aggressive progression than oligodendroglioma, and accordingly a diagnosis of an astrocytic tumor requires more aggressive therapeutic strategies. NOVA1 is an alternative splicing factor involved in the main mechanism of increasing proteome diversity coded by a limited number of genes. Together with other splicing factors, including ESRP1 and 2, MBNL1, PTBP1, and RBFOX2, NOVA1 contributes to establishing a cell type–specific splicing programs [26]. The role of altered expression of NOVA1 in glioma is still unknown. In oligodendrogliomas, Xu *et al*. [27] have reported the expression of multiple larger-sized transcripts for several genes attributed to hnRNP A1, a component of the spliceosome, which rules directly the selection of splice site leading to a preferential expression of larger-sized transcripts. These authors have suggested that the expression of large transcript could be useful for distinguishing oligodendroglial from astroglial gliomas [27]. In the present study, NOVA1 expression profile has proved to also differentiate oligodendroglioma from astrocytoma, and NOVA1 higher expression in oligodendroglioma may contribute to the preferential splicing program in this type of glioma.

In this work, we also detected and validated HSPB1 (HSP27) as a predictive factor for poor prognosis in GBM. High expression of HSPB1 was demonstrated in GBM cases with survival time shorter than 12 months. HSPB1 is a multifunctional protein that is dependent of oligomerization and phosphorylation status [28]. HSPB1/HSP27 is a human small heat shock protein, a chaperone that regulates fundamental cellular processes in normal unstressed cells as well as in many cancer cells, including breast, ovarian, endometrial cancers, osteosarcoma, and leukemia [29]. HSPB1/HSP27 is constitutively expressed at low levels in many cells and tissues, and its increased expression level has been correlated to the enhancement of cellular resistance, even in the presence of DNA damage due to UV radiation. Recently, the switch between apoptosis and survival, modulated by Akt stability, has been attributed to HSPB1/HSP27 in adenocarcinoma cells [30]. Interestingly, a network analysis by MetaCore™ has demonstrated that three targets, NPM1, GRP78 and RKIP, previously published by our group [4–6], are associated with the other two targets unveiled in the present combined analysis of proteomics and oligonucleotide expression arrays: HSPB1/HSP27 and nucleolin (NCL) demonstrated in Fig. 6. HSPB1/HSP27 is downstream of the canonical activation of EGFR through the transcription factor, nuclear estrogen receptor 1 (ESR1) [31]. Nucleolin (NCL) is a nucleolar phosphoprotein involved in the synthesis and maturation of ribosomes, found associated with intranuclear chromatin and pre-ribosomal particles, which induces chromatin decondensation by binding to histone H1. NCL plays a role in pre-rRNA transcription and ribosome assembly in the process of transcriptional elongation. NCL is downstream to RKIP in a canonical pathway, where RKIP down-regulates BRAF as demonstrated in melanoma cancer cells [32]. BRAF negatively regulates AKT [33] through directly binding to Rictor (mTORC2) [34]. AKT phosphorylates MYT1 kinase and decreases its activity [35], and the latter phosphorylates CDK1 and also decreases its activity [36]. CDK1 maintains NCL stability via phosphorylation during mitosis [37]. We have previously demonstrated that RKIP was down-regulated in gliomas [4], and therefore, RKIP would also determine down-regulation of NCL through this pathway. In contrast, NPM1 which is up-regulated in glioma [4] physically interacts with NCL and increases its activity [38]. On the other hand, HSPB1/HSP27 also up-regulates NCL, by binding to STAT3 [39], and ultimately, STAT3 may bind to NCL promoter [40], leading to cell proliferation. HSPB1/HSP27 may also activate NCL through SP1 [41], as NCL has been reported to physically interact with SP1 in non-small cell lung cancer [42], and human epidermal keratinocytes [43]. This NCL activation by HSPB1/HSP27 may receive reinforcement through GRP78, which physically interacts with ESR1 increasing its activity [44], which in turn also activates HSPB1/HSP27 promoter [45]. Therefore, HSPB1, GRP78 and EGFR may be linked through activation of two transcription factors, ESR1 and STAT3, and through a third transcription factor, SP1, this loop may be linked to RKIP, NCL and NPM1.

The present findings of HSPB1 expression enhancement with the tumor malignant progression, and NCL high expression in astrocytomas are in accordance with previously demonstrated differential expression of GRP78, NPM1 [4, 6] and EGFR [46] according to the malignant grade of astrocytomas, corroborating the relevance of this network in the progression of astrocytomas.

The combined methodology applied herein not only confirmed the relevance and the involvement of the molecular triad, NPM1, GRP78 and RKIP with glioma malignancy, but additionally has uncovered two new important targets: NOVA1, useful for the diagnostic refinement between astocytoma and oligodendroglioma, and HSPB1, as a predictive factor of poor prognosis for glioblastoma.

## Conclusions

Taken together, our quantitative proteomic analysis detected the molecular triad, NPM1, GRP78 and RKIP participating together with NCL and HSP27/HSPB1 in a network related to tumor progression. Additionally, two new important targets were uncovered: NOVA1 useful for diagnostic refinement differentiating astrocytoma from oligodendroglioma, and HSPB1/HSP27, as a predictive factor of poor prognosis for GBM.

## Additional files

Additional file 1:
**Figure S1 - Schematic experimental approach.**

Additional file 2:
**Figure S2 MetaCore symbol legend.**

Additional file 3:
**Table S1 Protein report.**

Additional file 4:
**Table S2 Peptide report.**

Additional file 5:
**Table S3 Protein ratio.**

## Abbreviations

ACN: Acetonitrile
AST II: AST II astrocytoma grade II
CHAPS: 3-[(3-Cholamidopropyl)dimethylammonio]-1-propanesulfonate
DTT: Dithiothreitol
FA: Formic acid
FDR: False discovery
GBM: Glioblastoma
GBM-LS: Glioblastoma, long survival, more than 16 months
GBM-SS: Glioblastoma, short survival, less than 12 months
GRP78: Glucose regulated protein 78 kDa
*GUS*: Beta-glucuronidase gene
*HPRT*: Hypoxanthine phosphoribosyltransferase 1 gene
HSP90B1: Heat shock protein 90 kDa
iTRAQ: Isobaric tags for relative and absolute quantitation
MS/MS: Tandem mass spectrometry
NCL: Nucleolin
NN: Non-neoplastic brain tissues
NOVA1: RNA binding protein nova 1
NPM1: Nucleophosmin
OLI II: Oligodendrogliomas grade II
OLI III: Oligodendrogliomas grade III
PCV: Procarbazin, 1-(2-cloroethyl)-3-cyclohexil-L-nitrosurea
qRT-PCR: Quantitative real-time PCR
RKIP/PEBP1: Raf kinase inhibitor protein
SCX: Strong cation exchange fractionation
SDS-PAGE: Sodium dodecyl sulfate polyacrylamide gel electrophoresis
*TBP*: TATA-box binding protein gene
Tris: Tris(hydroxymethyl)aminomethane)
