# Supplementary figures and images for: Quantitative proteomic analysis shows differentially expressed HSPB1 in glioblastoma as a discriminating short from long survival factor and NOVA1 as a differentiation factor between low-grade astrocytoma and oligodendroglioma
Source: BMC Cancer. 2015 Jun 25;15:481. doi: 10.1186/s12885-015-1473-9 (PMC4502388; doi:10.1186/s12885-015-1473-9)

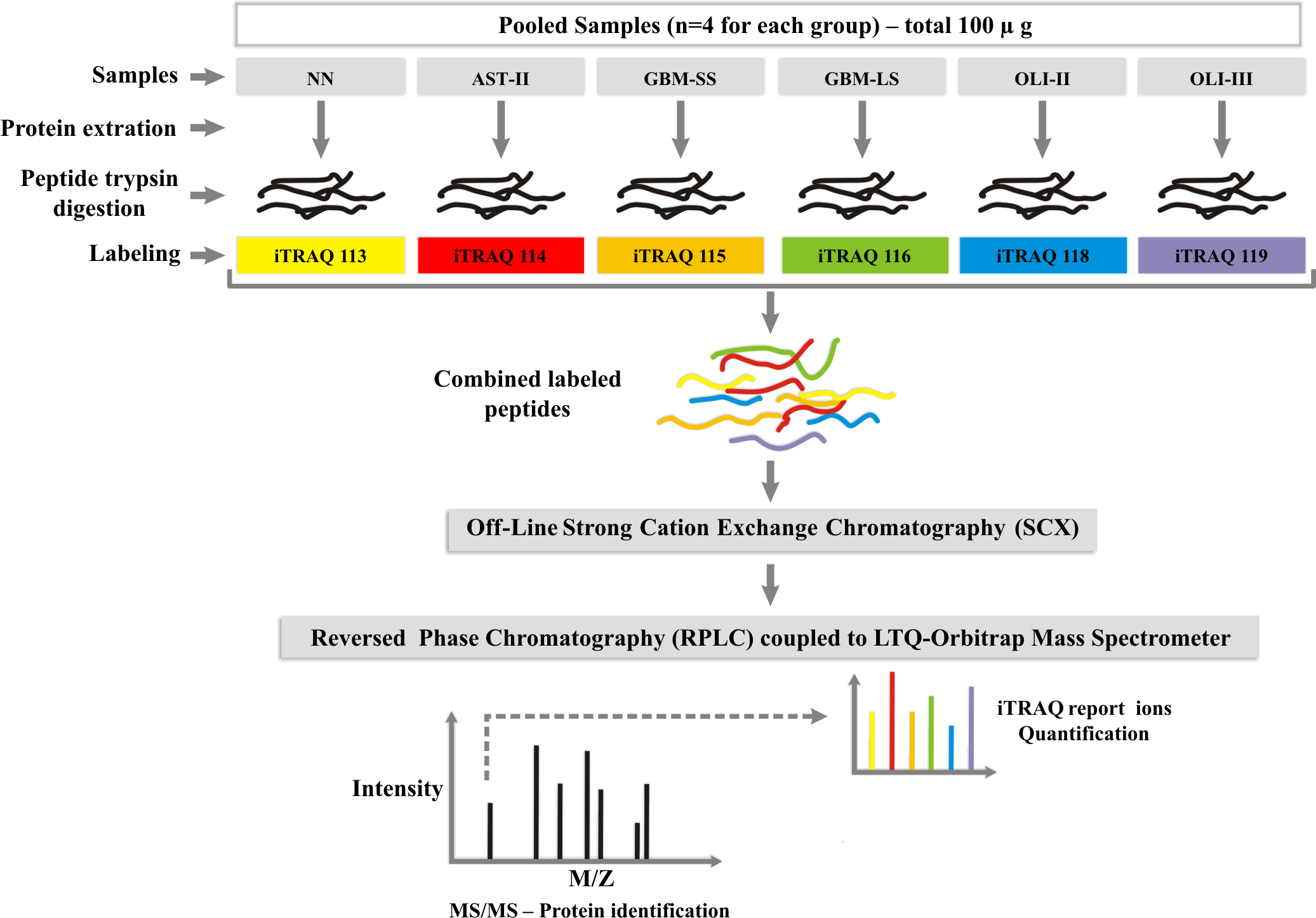

Supplement: Additional file 1: — Figure S1 - Schematic experimental approach. [file 12885_2015_1473_MOESM1_ESM.tiff]

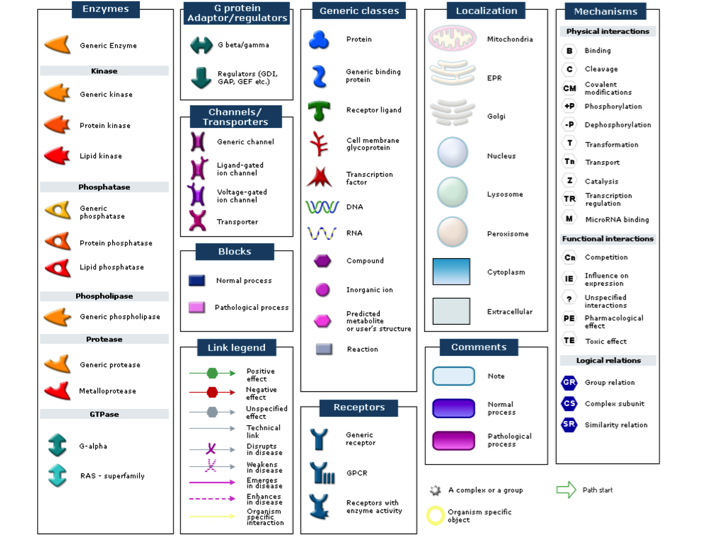

Supplement: Additional file 2: — Figure S2 MetaCore symbol legend. [file 12885_2015_1473_MOESM2_ESM.tiff]
